# Vested Interests in Addiction Research and Policy The challenge corporate lobbying poses to reducing society’s alcohol problems: insights from UK evidence on minimum unit pricing

**DOI:** 10.1111/add.12380

**Published:** 2013-11-21

**Authors:** Jim McCambridge, Benjamin Hawkins, Chris Holden

**Affiliations:** Faculty of Public Health and Policy, London School of Hygiene and Tropical MedicineLondon, UK1; Department of Social Policy and Social Work, University of YorkYork, UK2

**Keywords:** Alcohol industry, alcohol policy, corporate, lobbying

## Abstract

**Background:**

There has been insufficient research attention to alcohol industry methods of influencing public policies. With the exception of the tobacco industry, there have been few studies of the impact of corporate lobbying on public health policymaking more broadly.

**Methods:**

We summarize here findings from documentary analyses and interview studies in an integrative review of corporate efforts to influence UK policy on minimum unit pricing (MUP) of alcohol 2007–10.

**Results:**

Alcohol producers and retailers adopted a long-term, relationship-building approach to policy influence, in which personal contacts with key policymakers were established and nurtured, including when they were not in government. The alcohol industry was successful in achieving access to UK policymakers at the highest levels of government and at all stages of the policy process. Within the United Kingdom, political devolution and the formation for the first time of a Scottish National Party (SNP) government disrupted the existing long-term strategy of alcohol industry actors and created the conditions for evidence-based policy innovations such as MUP.

**Conclusions:**

Comparisons between policy communities within the United Kingdom and elsewhere are useful to the understanding of how different policy environments are amenable to influence through lobbying. Greater transparency in how policy is made is likely to lead to more effective alcohol and other public policies globally by constraining the influence of vested interests.

## INTRODUCTION

Alcohol industry actors may have commercial interests distinct from, and potentially at odds with, improving population health, yet they appear successful in positioning themselves as partners in policymaking processes in ways which would now be inconceivable for transnational tobacco corporations (TTCs) [1]. In comparison with TTCs, the alcohol industry has largely managed to avoid scrutiny of either the harms its activities cause or its attempts to influence public policies [2]. Although this has begun to change recently, there remains a need to develop the evidence base on when, how and with what degree of success alcohol industry actors attempt to influence the content of national and international policies.

Successive British governments have been strongly criticized for according industry interests too much weight in alcohol policymaking [3–6]. Consequently, it has been argued, alcohol strategies in the United Kingdom have been built around policies for which the evidence base is weak. These criticisms have largely stemmed from the contents of the policy documents themselves, although elsewhere industry involvement in policymaking has been successfully identified in other ways [7]. Investigation of the processes by which policies are made is necessary to determine whether and how far these criticisms are accurate and thus to provide a stronger basis for countering industry influence [8].

## METHODS

Investigating alcohol industry activities in the policy process involves greater methodological challenges than work on the tobacco industry, which is informed by internal industry documents made public as a result of litigation. These documents also provide information on the activities of parts of the alcohol industry, which have been found to work closely with TTCs [9],[10]. Research on the alcohol industry and other industries impacting public health and society usually relies upon publicly available documents, such as those submitted to government consultations, and interviews with key players in the policy process. The latter can involve both current and former ministers, members of parliament, civil servants, public health advocates and industry actors. These interviews can investigate the roles corporations play in the policy process, the extent to which their input is sought by government and the different avenues they pursue in order to represent their interests, as well as informing evaluations of the success of industry policy influencing activities. Such an approach is methodologically challenging as interviewees may not wish to reveal important information and/or seek to persuade the interviewer as to the interpretation of data. Careful triangulation of interviews between different respondents and with other data sources is thus required.

Following a review of the peer-reviewed literature, we examined formal policy documents and undertook a documentary analysis of industry submissions made to the Scottish Government's 2008 consultation on *Changing Scotland's Relationship with Alcohol* [11]. This was the first governmental publication within the United Kingdom to adopt a whole population approach to alcohol policy and as it was introduced by a minority government it was highly likely to be particularly contested. This study identified industry positions on policy proposals and investigated how research evidence was used in order to influence policy [11]. Subsequently, we completed 36 semi-structured interviews, 22 of which were with industry actors, defined as anyone directly involved in the production, supply or sale of alcohol [12] or in representing those interests. Respondents were identified through a stakeholder analysis [13],[14] and through snowball sampling, and included representatives from all sectors of the alcohol industry as well as senior politicians, civil servants and health advocates. Interviews were recorded, transcribed and analysed thematically using Nvivo software. A more detailed account of the methodology employed can be found elsewhere [15]. This paper offers an integrative review of our main findings.

## RESULTS

### Strategic positioning and framing policy debates

Debates about policy depend on the framing of the problem it is intended to address. Unsurprisingly, industry actors attempted to frame the impact of alcohol on society in keeping with their underlying corporate interests. They emphasized their economic importance (as employers and through tax revenue generated) and blamed sensationalist media for exaggerating the problems associated with their products [16]. Industry actors claimed that the majority of the population drink responsibly and that any new policies should be directed towards an allegedly small, problematic minority. They emphasized individual responsibility, calling for increased education and public information about harmful drinking [16]. This narrative is not new [17].

Somewhat paradoxically, rejection of whole-population interventions goes hand in hand with calls for a change in the drinking culture. The alcohol problem facing British society was framed predominantly in terms of binge drinking, not the mortality and morbidity caused by long-term heavy drinking [6],[18]. This framing invites a multi-sectoral approach led by criminal justice rather than health agencies, in line with the UK Government's current alcohol strategy [6],[19]. It also ignores the wider social problems created by alcohol, including the harms caused to children and families [20].

While policy preferences vary between individual companies, there is commonality in the positions adopted and significant capacity for collective action [15]. This began to change only when some form of price-based intervention began to seem unavoidable in Scotland [21]. The industry framing of policy debates was communicated both to policymakers and to the wider population through sophisticated media campaigns in an attempt to shape public opinion and the discursive environment in which policy decisions were taken [21].

Industry actors emphasized their corporate social responsibility (CSR) activities and their capacity for self-regulation, positioning themselves as part of the solution to alcohol-related harm, rather than the problem [22]. One former Public Health Minister described how partnership agreements are attractive to policymakers as they avoid the costly and time-consuming processes of passing and enforcing legislation [23]. Where self-regulation is impossible, industry actors seek to integrate themselves into the policy process so that policies are co-produced by policymakers and corporate actors [24].

Industry actors claimed to be committed to evidence-based policy [11], yet they consistently opposed the international research community consensus that the policies most likely to be effective in reducing alcohol-related problems in the population are increases in the price of alcohol, and restrictions on availability and marketing [25]. Documentary submissions to the Scottish Government consultation failed to engage with the research literature in any depth, although made no shortage of claims about it. This and other tactics in relation to evidence are used by industry actors elsewhere [11],[26–28]. Strong evidence was misrepresented and weak evidence promoted when comparisons were made with the expert summary of the peer-reviewed literature [25]. Unsubstantiated claims were made about the adverse effects of unfavoured policy proposals and advocacy of policies favoured by industry was not supported by the presentation of evidence [11].

### Policy influencing activities

Lobbying, by the alcohol industry and other sectors, is extensive. According to David Cameron, before he became UK Prime Minister: ‘We all know how it works. The lunches, the hospitality, the quiet word in your ear, the ex-ministers and ex-advisers for hire, helping big business find the right way to get its way’ [29]. A recent judicial inquiry (by Lord Leveson) into the culture, practices and ethics of the UK press has drawn attention to lobbying via ‘the indefatigable use of text messaging, email and telephone’ (p. 1376 in [30]). Such views of lobbying are widespread, yet there are few rigorous research studies of lobbying on alcohol or other public health issues.

Key findings from our investigations of lobbying are presented in Box 1 [23]. The dominant approach used by the alcohol industry was to nurture and sustain long-term relationships with policymakers, within which subtle forms of influence were exercised. This reinforces, and is reinforced by, the industry narrative that they are key stakeholders in the policy process whose voices should be heard. Where these long-term relationships fail to secure a favourable regulatory environment, however, industry actors will lobby key decision-makers forcefully on an issue by issue basis, including both government Ministers and opposition MPs/MSPs [23]. Where this proves unsuccessful, they will pursue their interests through other means, including threatening and conducting legal challenges under national and international law. This underlines a highly pragmatic approach to policy influence in which long-term relationships are favoured, but where the partnership approach is abandoned if circumstances demand it.

**Box 1 Lobbying in the United Kingdom 2007–10: alcohol industry actors**

- Took a long-term relationship building approach with key decision makers, based on the provision of assistance and information and the promise of delivering policy outcomes through co-and self-regulatory regimes;
- engaged Members of Parliament from all parties, civil servants, Ministers, Shadow Ministers and special advisers through a range of different channels, including party conferences and Parliamentary All Party Groups;
- enjoyed considerable access to policymakers at all stages of the policymaking process, attending regular meetings throughout the year;
- benefited from extensive informal personal contacts in government;
- were consulted informally both before and after official policy consultation events;
- were thus successful in positioning themselves as key stakeholders in the policy process, who must be consulted on policy developments as a matter of course.

Further evidence of this pragmatism lay in the ability to co-ordinate policy influencing activities, despite the commercial rivalries which exist [15]. Industry actors often sought to influence policy though trade associations, although the largest corporations also represented their own interests independently [15]. In Scotland the campaign against MUP was led by the Scotch Whisky Association, primarily to take advantage of the particular economic importance of the whisky industry in Scotland and the status of its product as a cultural icon [21].

Considerable time and other resources are expended by industry actors in the ‘proactive influencing’ of policy, as the Portman Group described it [23]. The personal relationships cultivated are central to the success of these influencing efforts [23]. Industry influence varies, partly because policy environments facilitate the fostering of these relationships in different ways and to different degrees. Comparisons between Westminster and Edinburgh are instructive in this regard—see Box 2 [21]. In the latter, a greater culture of openness and willingness to engage with stakeholders has been observed [31],[32]. The election for the first time since devolution of a Scottish National Party (SNP) government not associated with the Westminster political parties made MUP more attainable [21]. The level of access afforded to public health advocates by the new administration facilitated a reframing of the policy debate and an openness to whole population interventions such as MUP. Crucially, it disrupted the existing, long-term relationships developed by the alcohol industry with the UK-wide parties previously in power. The industry approach of focusing their lobbying efforts on these parties was successful until the SNP gained a parliamentary majority in 2011 and made MUP a priority.

**Box 2 How minimum unit pricing (MUP) gradually gained support in the United Kingdom despite alcohol industry opposition**

- The election of the minority Scottish National Party (SNP) administration in 2007 disrupted longstanding relationships between industry actors and Scottish Ministers.
- Despite continued access to decision-makers by industry actors, public health advocates helped the Scottish Government change the terms of the debate on alcohol problems and their solutions.
- Industry lobbying adapted to the political landscape, focusing instead on opposition Members of the Scottish Parliament, backed up by an extensive media campaign against MUP.
- These tactics succeeded initially in stopping the passage of the measures through the Scottish Parliament in 2010 but they were later passed by a majority SNP Government after the 2011 Scottish Election.
- The SNP's policy moved MUP up the agenda elsewhere in the United Kingdom. It was later included in the UK Government's alcohol strategy for England, although not implemented.
- Industry actors see UK policy as particularly important for the effects it could have on regulation and corporate strategy in emerging markets such as China, undermining arguments for market liberalization there.
- Implementation of MUP in Scotland has been delayed by industry legal challenges.

## DISCUSSION

This work confirms that transnational alcohol producers and the large supermarkets have access to policy actors in the United Kingdom at all levels of government and throughout the policy process, including to opposition parties. A Public Health Commission (PHC) established by the Conservative Party in opposition during the 2007–10 study period formed the basis of the Public Health Responsibility Deal which became central to the subsequent UK government's public health policy [33]. This institutionalizes the role of industry actors in UK public health policy in an unprecedented way, enhancing capacity to shape policy formulation and to deflect or delay policies that are contrary to these vested interests.

Such developments call for a deeper understanding of corporate strategy and tactics to investigate further how this access is used to influence policy, and to assist the development of better protection against the harms caused to society by alcohol. The tobacco industry is well established as a pioneer of corporate political strategies [34], and much is now known about their activities through court mandated disclosure of internal industry documents in the United States. The tobacco and alcohol industries are also not dissimilar, in that both are involved in the legal production, distribution and retail of a drug which is toxic, addictive and causes high levels of death and disease (each being responsible for approximately 5–6% of the total global burden of disease [35]). They also have histories of working together to avert policy measures with capacity to address this situation (for example on pricing and promotions) [9] and patterns of co-ownership continue to exist [10],[36].

Lessons learned about TTCs may provide new foci for the study of corporate influence on public policy by actors from other industries. For example, the stated goals of industry CSR activities may differ greatly from corporations' real aims and objectives. These may include attempts to gain the legitimacy and access to policy-makers which facilitate long-term relationship-building [37]. Alcohol industry actors will also have learned from the experience of TTCs, and it is interesting how closely aligned in the United Kingdom between 2007 and 2010 were the producers and the big supermarkets in their efforts to influence policy.

Similarly, where industry interests are significantly harmed, or the partnership approach is rejected by policymakers, alcohol industry actors have demonstrated they are prepared to deploy more confrontational tactics of the kind now routinely used by TTCs. This has been seen in the period since our study. These include legal challenges to policies, not only under domestic law, but at European and global levels [e.g. disputes within the World Trade Organization (WTO) for apparent violations of trade law] [38],[39]. The French *Loi Evin* restricting alcohol advertising was tested earlier at European Union (EU) level [40] and the UK and European arms of the alcohol industry have mounted legal challenges to MUP under EU single-market law [41]. Appeals under European law have the potential to delay the implementation of MUP in Scotland (and by extension the rest of the United Kingdom) for many years.

Thailand's attempts to introduce graphic warning labels on alcohol containers have been opposed by WTO members, including the EU, as unnecessary restraints on trade since 2010, in very similar ways to how Australia's cigarette plain packaging laws have been contested [42]. Such legal challenges and trade disputes may be mounted even when the companies concerned know that their arguments have little basis in law [43]. The purpose is often twofold: to exert a ‘chilling effect’ on other governments considering similar legislation and to delay the implementation of legislation in the targeted country, during which period the companies concerned can continue to promote their products without the hindrance of the impending regulation. The time and expense necessitated by such legal cases, while affordable for large transnational corporations, can be a very real deterrent for governments, especially those of low-income countries.

It is obvious that tensions with policymakers may arise when profit is pursued through the sale of products which cause health or social problems [28]. The handling of these tensions is a core component of corporate strategy [44–46], and lobbying is an important centrally directed activity in large corporations [24]. The pragmatism we identified provides further evidence that the particular tactics employed by corporate actors develop over time in relation to both the degrees and types of regulation being considered by policymakers and the policy environment in question [46]. At times, this may include attempts to shift decision-making to venues—at the subnational, national or supranational levels—in which they are most effectively able to influence policy. In Scotland, attempts by certain actors to refocus debates onto taxation as opposed to MUP can be seen as an attempt to shift policymaking to Westminster (where tax policy for the entire United Kingdom is set), as it was felt that the government there was more sympathetic to commercial interests [21],[23]. In addition, corporate strategy adopted in one location may also be influenced by global considerations rather than domestic priorities. Opposition to MUP in Scotland appeared to be driven as much by concerns about its impact on attempts to gain market access and ensure favourable regulatory environments in emerging economies as by domestic concerns [21].

These considerations indicate the potential value of developing conceptual frameworks that will guide further study. For example, the political science literature on multi-level governance [47] appears to have clear relevance to the study of the alcohol industry. Jahiel [48] and colleagues [28] posit a more epidemiologically based model in which corporate profit-making is linked to individual health and social consequences, that has been applied to alcohol. In both cases, specific lobbying activities can be understood or situated within larger corporate political strategies.

The ability of policymakers to manage corporate influence in the public interest is profoundly challenged by globalization. According to Casswell [1]: ‘the global alcohol industry is engaged in a race against time, to ensure the diffusion and normalization of drinking in emerging markets before governments and civil society are able to ensure adequate policies to limit the spread of heavy drinking and alcohol-related harm in the population’. An analysis of four draft national alcohol policy documents in sub-Saharan African countries found them to be almost identical in wording and structure and implicated one particular corporation (SAB-Miller) in their construction [7]. The policy documents included promotion of the health benefits of alcohol, which are unlikely to occur widely in countries where average life expectancy is lower than the ages at which any such benefits may be expected [7]. The potential contribution to global health that high-income countries can make by addressing vested interests should not be underestimated.

Corporate lobbying presents formidable challenges for public policies world-wide and comparative analyses of industries and countries will be important, as well as studies of the changing policy contexts faced by the alcohol industry over time within a given country. Corporate influence is exercised through many channels. Our study suggests that personal relationships established and nurtured over the long term are key and they are utilized at all stages of the policy process, including at the highest levels of government by corporations with vested interests in policy outcomes. Our investigations suggest that there is much more work to be carried out in uncovering the mechanisms, as well as establishing the extent, of corporate influence on policies within as well as between countries. It is unclear how the apparent success of corporate influence on alcohol policy in the United Kingdom [6],[19] compares with other areas important to health, such as food. As Lord Leveson put it, corporate lobbying entails ‘placing the conduct of public policy issues outside the mechanisms of transparency, accountability and public record’ (p. 1405 in [30]). As such, it ‘cannot but give rise to perceptions and questions which are corrosive to public trust and confidence’ (p. 1405) [30]. This provides a very useful guide to what public policymakers across the world should do about corporate lobbying. Transparency in all aspects of lobbying, including money spent on it, should be a key issue for alcohol policy reform. Public policy will benefit from careful studies of how policy is actually made, and the support of policymakers is essential for this. We also need more assertive promotion of the ideas that protecting and promoting population health and the interests of society are core duties of government, if effective alcohol policies are to be introduced.
